# Factors Associated with Willingness to Receive a COVID-19 Vaccine in Adult Polish Population—A Cross-Sectional Survey

**DOI:** 10.3390/vaccines10101715

**Published:** 2022-10-14

**Authors:** Katarzyna Ulaszewska, Alicja Monika Jodczyk, Piotr Długołęcki, Sara Emerla, Wiktoria Stańska, Przemysław Seweryn Kasiak, Jakub S. Gąsior, Damian Parol, Artur Mamcarz, Daniel Śliż

**Affiliations:** 1 Students’ Scientific Group of Lifestyle Medicine, 3rd Department of Internal Medicine and Cardiology, Medical University of Warsaw, 04-749 Warsaw, Poland; 2 Polish Society of Lifestyle Medicine, 00-388 Warsaw, Poland; 3 Department of Pediatric Cardiology and General Pediatrics, Medical University of Warsaw, 02-091 Warsaw, Poland; 4 3rd Department of Internal Diseases and Cardiology, Medical University of Warsaw, 04-749 Warsaw, Poland; 5 School of Public Health, Postgraduate Medical Education Center, 01-813 Warsaw, Poland

**Keywords:** vaccinations, COVID-19, SARS-CoV-2, Poland, public health, vaccine hesitancy

## Abstract

Vaccinations are proven to be the most efficient in preventing COVID-19 disease. Nonetheless, some people are skeptical and hesitant. The study aimed to determine factors associated with willingness to receive a COVID-19 vaccine in the Polish adult population. An online survey consisting of questions regarding (1) demographic information and (2) health issues (the status of vaccination, comorbidities, receiving the flu vaccine and usage of health monitoring apps) was distributed between 13 January and 14 February 2022. Of the 7018 participants who met the study conditions, 76.89% (*n* = 5396) were females, 22.44% (*n* = 1575) were males and 0.67% (*n* = 47) did not specify gender. The median age was 31 years. Among them, 81.82% (*n* = 5742) were vaccinated and 18.18% (*n* = 1276) were not. 46.87% (*n* = 3289) had no chronic co-morbidities. Factors associated with lower odds to receive the vaccine were: being men (*p* = 0.02; OR = 0.83), having lower education status (*p* = 0.001, OR = 0.56–0.77), living in a smaller residence area (*p* < 0.001, OR = 0.47–0.73.), not receiving flu vaccination (*p* < 0.001, OR = 24.51) and not using health monitoring applications (*p* < 0.001, OR = 1.56). Health education and communication strategies are needed to achieve large-scale vaccine acceptability and finally herd immunity.

## 1. Introduction

The COVID-19 pandemic and the imposed restrictions have had a significant negative impact on the main areas of health. There have been harmful consequences to sleep and mental health [1], diet and physical activity [2], drug usage and the risk of depression [3]. Moreover, those effects of the pandemic and the following restrictions have been observed not only among healthy and young individuals but also among those with various diseases [4]. Vaccination is one of the best methods to prevent the spread of the SARS-CoV-2 virus [5], yet the healthcare sector continues to suffer from a high percentage of unvaccinated people. One of the factors hindering proper vaccination of the population is disinformation caused by the spread of fake news by non-professionals [6].

Vaccine hesitancy is a worrying phenomenon due to its range and health-related consequences [7]. In Poland the number of mandatory childhood vaccination refusals increased from 3437 to 48,609 in 2010–2019 [8]. Vaccination refusals may be associated with the growing activity of anti-vaccination movements. The popularity of social media and access to the Internet enables anti-vaccination movements to spread their views [8,9]. Other reasons for vaccination refusals can be secondary to a lack of appropriate education in this area, especially by medical personnel [8,10]. The study conducted in 2019 among Polish citizens showed that at least 85% of those surveyed believed that childhood vaccination helps improve children’s health and 73% were convinced that vaccination is safe for children [8], which could act as a proxy for the present pandemic situation. Regarding this information, it is surprising that at the time of the study, people in Poland who were fully vaccinated against COVID-19 accounted for only 56.62% (*n* = 21,400,739) [11] of the population, yet vaccines had been available free of charge to every adult since 10 May 2021 [12]. The state of the Polish population in the summer of 2021 was 38,080,000, of which 81.8% were adults [13]. The number of confirmed infections on the first day of the study (13 January 2022) was 16,878, and the highest number observed during the survey was (on 27 January 2022) 57,659 confirmed infections [11]. There were restrictions on the wearing of face masks and limitations on people staying in public places (some limits did not include people with a current COVID certificate), and classes took place in hybrid form.

Awareness of the motivations and factors behind the COVID-19 vaccine refusal constitutes essential information for public health and healthcare professionals as many people remain unvaccinated and the situation may repeat in the future because of another pathogen. Additionally, the Polish adult population serves as an example of an environment with distinct social conditions present—also for vaccinations—in European post-socialist countries. [14] The current literature in the field demands further analysis on the reluctance towards anti-COVID-19 vaccines in new democracies of Central and Eastern Europe.

The aim of our study was to determine factors associated with willingness to receive a COVID-19 vaccine in the Polish adult population. 

## 2. Materials and Methods

### 2.1. Study Design and Instrument

**Figure 1:**
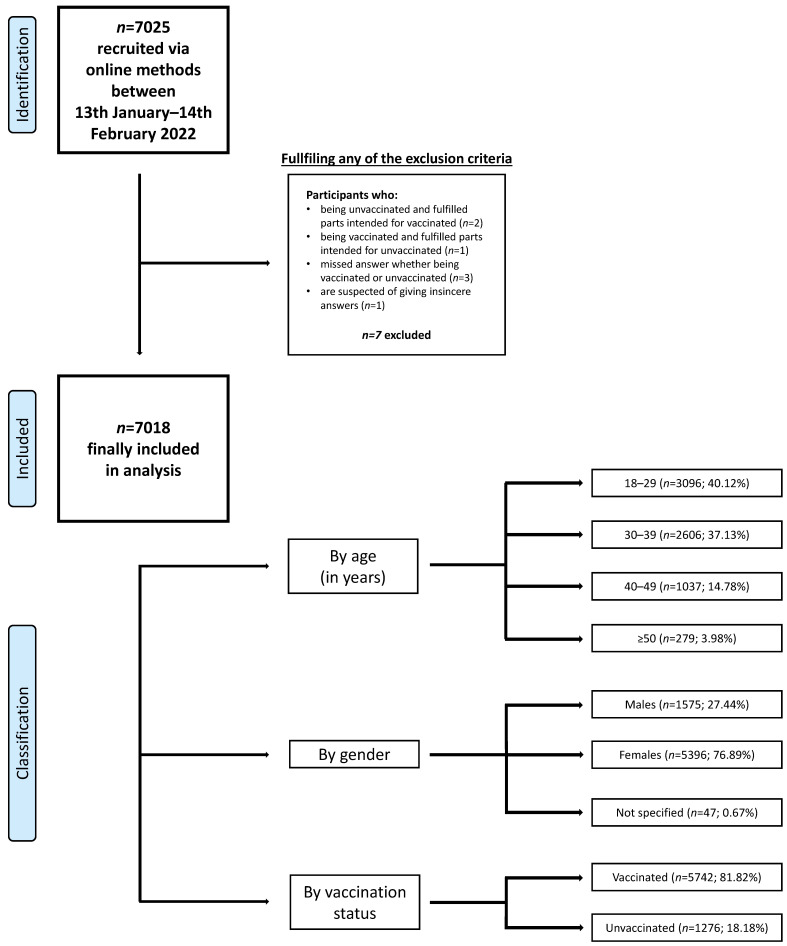
Data selection process.

The study was conducted between 13 January and 14 February 2022 among Polish citizens. At that time, the number of COVID-19 infections in Poland, referred to as the “fifth wave” of the pandemic, was increasing. Of the 7025 adults who participated in the study, 7018 met the study conditions. To be included in the study, the respondent (1) had to be at least 18 years of age (2) and be able to complete an online survey. Exclusion criteria included: (1) fulfilling questions in the wrong category, (2) responses with a high suspicion of being insincere (such as person under 30 with 10 or more children) and (3) omission of the answer to the question about vaccination status. Respondents were classified into groups based on their gender, age and vaccination status. The full participants selections process is presented on the Figure 1.

The study was performed by an online survey which was designed and shared via different social media channels (e.g., Polish Society of Lifestyle Medicine newsletter, students’ university groups, Instagram, Facebook and LinkedIn). We chose the form of an online survey in order to reach a large and diverse group of respondents and reduce face-to-face contacts. The minimum sample size was calculated by the sample size formula for cross sectional studies and survey studies. A selected reference sample was chosen (*n* = 371). The following variables were applied for the calculations: standard normal variate (5%), type I error, expected proportion in the population (59% of vaccinated Polish population) and absolute error (5%).

The survey consisted of four main parts: (1) general information: gender, age, marital and educational status, working activity, place of living and having children, (2) information regarding one’s health: chronic diseases, self-rated lifestyle and health status, receiving the flu vaccine in 2021 or 2022, usage of application monitoring health. The entire survey was formed by selecting the following questions by a team of experts comprising an internal medicine physician, a specialist in public health, a lifestyle medicine practitioner and an environmental epidemiology professional. Most of the questions in our survey were based on the validated questionnaire included in the study conducted by Polla et al. (2021) [15], which was tested on a group of 50 people in a pilot test. Information regarding marital status, education, overall health assessment and having chronic comorbidities are typical questions characterizing the population, and also were a part of the mentioned study. Questions about receiving the flu vaccine were obtained from the article written by Fisher et al. (2021) [16], where the questionnaire was validated by conducting the pilot test in 2 rounds. The study conducted by Paradis et al. [17] inspired questions about using health monitoring applications in our study. The question related to overall-self reported health assessment is the same as in the 2002 World Health Survey [18], and is also used by the World Health Organization. The question regarding the overall-self reported lifestyle assessment is an author’s question.

The questionnaire form is available in the Supplementary Material (S1—Questionnaire form).

### 2.2. Ethics

Institutional Ethics Committee review and approval were waived for the presented study because it was not a medical experiment, it only involved anonymous data and was conducted online. According to the act of 5 December 1996 on the professions of physicians and dentists ([19]; no ethical consent is required). The study is in line with the Institutional Ethics Committee of the Medical University of Warsaw regulations and the Helsinki Declaration (1964). Participants did not receive any financial or material benefits from answering the questions. The information about anonymity and a detailed study description was stated at the beginning of the questionnaire. Fulfilling the form was obtained as consent to participate in the study.

### 2.3. Data Analysis

Basic data were saved into an Excel file (Microsoft Corporation, Washington, DC, USA). Categorical variables were presented as a sample percentage (%). The differences between groups were verified by a Chi-square test for categorical data or, due to non-parametric distribution (confirmed by using the Shapiro–Wilk test), for continuous data by the U Mann–Whitney test. Binary logistic regression was employed to identify factors associated with the outcome variable. Model fitness was checked by using the Hosmer–Lemeshow goodness of fit test. To express the performance of the logistic regression models, the area under the curve (AUC) statistic was used. The two-sided significance level *p* ≤ 0.05 was considered as significant. Analysis were performed using Statistica 13.3 software (TIBCO Software Inc., Palo Alto, CA, USA).

## 3. Results

### 3.1. Sociodemographic Characteristics

A total number of 7025 respondents participated in the study, and 7018 of them met all the inclusion criteria (5396 females, 1575 males, 47 did not specify gender; median age 31 years, range: 18–75). Among them, 81.82% (*n* = 5742) were vaccinated and 18.18% (*n* = 1276) were not. Demographic characteristics of the participants, including gender, marital status, education status, occupation, residence area and number of children are shown in Table 1, with a distinction between vaccinated and unvaccinated participants.

Among those surveyed, 82.77% female, 78.48% male and 85.11% who did not specify their gender, were vaccinated against COVID-19. In the age category, it was classified as follows: 80.04% (*n* = 2478) of people aged 18–29, 82.16% (*n* = 2141) of respondents aged 30–39, 85.44% (*n* = 886) people aged 40–49 and 84.95% (*n* = 237) people aged 50 and over were vaccinated.

### 3.2. Chronic Co-Morbidities

Among all respondents, 3289 (46.87%) were apparently healthy (no chronic co-morbidities)—78.20% of them received the vaccination; 3729 (53.13%) had at least one chronic disease and 85.01% of them were vaccinated. The prevalence of chronic diseases among respondents is shown in Table 2. In the group of chronically ill responders, the highest vaccination rate was in patients with hypertension, obesity and psychiatric conditions, achieving 88.70%, 87.77% and 88.20%, respectively.

### 3.3. Factors Associated with COVID-19 Vaccination

In order to identify factors that could determine COVID-19 vaccination, multivariable logistic regression was applied.

The category with the higher number of participants was chosen as the reference group (odds ratios will be a comparison to the reference group). Gender, education, occupation, residence area, chronic comorbidities, use of health monitoring applications and receiving the flu vaccine in previous years were found to be significantly associated with the COVID-19 vaccination.

Males, participants with lower education status from smaller residence areas who did not have chronic comorbidities, those who did not use health monitoring applications and those who did not receive the flu vaccination in previous years had lower odds of COVID-19 vaccine uptake.

The Hosmer–Lemeshow goodness-of-fit test produced a test statistic of 5.698 (with a *p*-value of 0.68). AUC of the regression model was 0.729.

For detailed information about multivariable logistic regression please see the Table 3.

Results of the multivariable logistic regression without receiving the flu vaccine are presented in the Supplementary Materials (S2—Table S1. Multivariable logistic regression analysis of factors associated with COVID-19 vaccination.).

## 4. Discussion

The main findings of the study are the characteristics of respondents least likely to vaccinate: males, people with lower education, those living in smaller towns and villages, those not suffering from chronic diseases, those who do not use health monitoring applications and also those who are not vaccinated against the flu. People with identified determinants should receive extra attention while planning and performing mass-scale vaccination.

We managed to reach the data from the representative sample of 70,195 adults, while approximately 31 million of them live in Poland. That number of participants provides an image of the population. There are no studies concerning COVID-19 vaccination hesitancy on such a large number of Polish individuals, apart from persuasive messages studied by Kachurka et al. [20]. Previous studies conducted on Poles available to the authors, which regarded willingness to vaccinate or take a booster dose or reasons for hesitation, were conducted on smaller groups consisting of 1000–2500 participants [21,22]. The novelty of this study is the collection of proportional groups of respondents in terms of the prevalence of chronic diseases (46.87% of healthy people, 53.12% of chronically ill people), as well as the determination of which diseases influenced the decision to vaccinate against COVID-19 among the respondents. Another value not described before is the influence of using health monitoring applications on the choice of vaccination. Our research has the potential to draw the attention of clinicians and caregivers, raise awareness of the factors connected with lower odds to vaccinate and determine the group of the highest concerns.

We describe the factors connected with vaccinations after a nearly two-year pandemic period in Poland, while the vaccine program was available to everyone. Therefore, our results indicate the consequences of recurring lockdowns, prolonged social limitations and government campaigns to promote vaccination. Vaccine hesitancy encompasses a spectrum of attitudes towards vaccinations between full acceptance and total refusal. Its determinants are multifactorial and may be divided into three groups of influences—contextual impacts (e.g., historic, economic), individual or group perceptions and vaccine-specific effects. From such a perspective, health communication only acts as a modulatory element, not a determinant itself [23]. The origin of vaccine hesitancy dates back to the times of Edward Jenner [24]. The COVID-19 pandemic has presented new challenges in this field. Among others, rapid development of different preparations, including new public opinions on mRNA technology, became a background for many suppositions [25]. This especially relates to the social media domain as everyone can publish their own opinions. Numerous statements from individuals without proper knowledge and medical education lead to wide misinformation in the area of COVID-19 vaccines [26].

In Poland, widespread vaccination, besides the current pandemic situation, only applies to children [27]. By the beginning of 2022, over 21 million Poles had been completely vaccinated (one dose of single dose vaccine or a second dose of two dose vaccine) against SARS-CoV-2 [28], a little more than half of the population. It would be interesting to parallel this data with the statistics for another recommended vaccine against infectious disease—influenza, yet its usage is scarce in Poland—less than 3% out of nearly 40 million people, mostly elderly adults [29]. Societies of the post-socialist countries (such as Poland), as members of the European Union tend to have lower rates of immunization against SARS-CoV-2 than older democracies. It has been indicated that trust in authorities and in informal sources of information may serve as key explanations [14,30]. Checking the potential acceptance of immunization against COVID-19 at the beginning of the pandemic revealed that Polish respondents gave the most negative answers (27, 3%) out of 19 nations (*n* = 13,426) in terms of taking a “proven, safe and effective vaccine” [31].

Although males are more susceptible to SARS-CoV-2 infection and the severe course of COVID-19 [32], our study shows that they are less likely to be vaccinated. Similar findings were obtained from France, Germany, Sweden, Russia [33] and Romania [34]. The opposite results for males were presented for the Polish population on a similarly large sample of adult Internet users. [20] Otherwise, gender was found to be insignificant in cross-sectional studies of Hungarian [35] and a Croatian populations [36]. However, an international systemic review and meta-analysis of predictors linked to acceptability, where among socio-demographic factor gender and educational level were found to be the most efficient, linked females with higher hesitancy [30]. Our results and conflicting findings in the literature demand further attention as to possible regional differences between genders in vaccine hesitancy and their foundations.

Furthermore, being older or better educated was also associated with higher COVID-19 vaccine acceptance in Poland [20]. Results of our study confirm the influence of educational status but demonstrate no significant effect of age (however, among respondents, older people were more often vaccinated than young adults). That is in agreement with the mentioned systemic review and meta-analysis, which also highlights the role of trust in the government and influenza vaccine history as strong predictors [30]. Thus, we can add more evidence to international results. In our case, occupation acts as a confounding factor, because we found those who were unemployed to be more willing to vaccinate, which is in contrast to global COVID-19 vaccine hesitancy [37].

In our study, being vaccinated against the flu in the last season significantly predisposed people to being vaccinated against COVID-19. Similar relationships were found in Hungary [35] and northern Slovakia [38], as well as in several other studies concerning different populations and health care workers [39,40,41] or students [42]. Because of its disadvantages [43], we see influenza vaccination as an indicator of vaccine acceptance, vaccine literacy and even vaccine enthusiasm. Significant agreement of scientific research, including our work, allows to perceive vaccinations against flu and COVID-19 as deeply related. Therefore, we suggest that it is advisable to develop and use a combined preparation.

Residence area also mattered in our research—living in smaller settlements was associated with a lower probability of immunization, which was also true for the Croatian population [44]. Reverse dependence was observed in Hungary, although the difference became insignificant when capital city was compared to villages [26]. It is worth noticing that in a vast study of nearly half a million responses from Latin America and the Caribbean, results supported higher vaccine intention in cities than in rural areas [45]. Hence, inhabitants of smaller settlements in Poland, as well as in many other countries, may require additional vaccine-promoting strategies.

COVID-19 disease is especially dangerous for people who suffer from chronic diseases. Research and statistics show that people with chronic diseases are more often hospitalized due to SARS-CoV-2 infection than healthy people, even among children [46]. The most common chronic diseases that cause hospitalization are obesity, cardiovascular diseases and hypertension [47,48,49]. Likewise, these diseases can be associated with severe infection as well as complications after recovery [50,51]. It turns out that severely obese patients produce reduced antibody titers and should therefore be prioritized in the COVID-19 vaccination [52]. Surveys show that chronically ill people are more positive about vaccinations against influenza or pneumococcal disease [53] and are more willing to vaccinate their own children against COVID-19 [54]. Similar to the findings of our study, a survey of public opinion in Australia before the start of COVID-19 vaccination demonstrated that people with chronic diseases declared more willingness to take the vaccine [55]. Our findings, supported by the literature, possibly indicate higher vaccine literacy or fear of COVID-19 in this subpopulation.

The global results also show that people in relationships are more likely to vaccinate compared to those who were single [37]; in our study, it turned out to be statistically insignificant compared to marriage.

Applications for controlling health are becoming more popular and they may soon help reduce health care costs [56,57] or even support the treatment of chronically ill people [58]. Using the application affects the patients’ motivation to take care of their health and brings effects, e.g., in the form of increasing physical activity [59]. More pleasing is the fact that people who care about their health more often decide to vaccinate against COVID-19 in Poland. Our research explores this novelty, which is understudied in current literature. Similar to the flu vaccination, it reveals that interest in one own’s health determines SARS-CoV-2 immunization. We see the development of trusted mobile health applications as an interesting direction for decreasing vaccine hesitancy.

As may be concluded, identified factors influencing vaccination decisions in Poland, a European post-socialist country, largely resemble worldwide determinants. Literature would benefit from multinational studies, e.g., covering the region of Central and Eastern Europe, to precisely target vaccine promotion [60]. More in-depth analysis might explore trust in information sources and knowledge about COVID-19. They represent confounding factors in the decision making process on immunization against SARS-CoV-2 and may influence determinants found in this study. On the example of older people, it has been proposed that understanding the knowledge, views and behaviors of the vulnerable population can significantly help to reduce the scale and burden of the COVID-19 pandemic [61]. However, it is not universal for all patient subgroups [62]. Self-perceived knowledge requires a distinction from actual knowledge as less reliable, what was demonstrated among Romanian oncological patients [63]. Higher scores in the COVID-19 knowledge test were positively and independently associated with vaccination, as well as respecting other preventive measures [64].

## 5. Practical Implications

The study shows how divided society remains about the decision to vaccinate against COVID-19. It is especially important to reach out to and educate people who remain unvaccinated, as 70–80% total vaccination status must be achieved in order to be considered immunologically safe [65]. The survey results are mainly addressed to healthcare professionals and public health specialists. The above-mentioned professions constitute a pillar of health education and may influence the flow of information and health promotion. It is important that patients are adequately educated about the SARS-CoV-2 pandemic and that if a similar situation repeats itself in the future, they know how to behave and what they can do to avoid infection effectively. Then there should be social campaigns aimed at people who are particularly skeptical, using the popularity of the Internet and social media, spreading medical knowledge, limiting fake news and warning against them. It is crucial that patients trust and feel safe. Understanding the determinants of people’s reluctance to get vaccinated can be a useful tool for designing actions to increase awareness and vaccination coverage, especially against COVID-19. Data identifying predictors of vaccine hesitancy can be used to guide SARS-CoV-2 vaccination promotion policy, increase base compliance and booster doses and point out weaknesses in the implemented strategic measures.

## 6. Further Studies Directions

The validation of the survey was not the aim of our study. We recommend using our questionnaire in subsequent works and its validation. For a more comprehensive view on the situation, more profound research should be performed in the future [13].

## 7. Limitations

Several limitations should be considered when interpreting the results. The presented study was conducted online [66]. We cannot be certain that the responders fully understood the questions and answered them correctly. The whole data, including the status of vaccination, was self-reported, and we cannot be sure whether they are reliable. The question about the status of vaccination did not separate fully vaccinated respondents with those with incomplete vaccination. In addition, we did not consider the possibility that failure to vaccinate was related to contraindication to vaccination and not with unwillingness. The questionnaire was mostly based on authors’ questions regarding demographic data and therefore was not validated. Thus, our results should be interpreted carefully. The sample was relatively disproportionate and we managed to recruit a comparatively higher number of females than males and a higher number of respondents with a higher education level than with a lower education level. Therefore, more cross-sectional research on wider populations should be performed.

Prognostic models do not always work in practice; therefore, their validation is recommended; however, they are used in medicine to test a patient’s treatment outcomes in relation to patient and disease characteristics [67]. Such data can be used to introduce changes to older regulations [68]. The use of a tool such as a non-validated survey depends on the context and the surveyed population, may be affected by a measurement error and conclusions may be incomplete [69].

## 8. Conclusions

Summarizing the presented results, the study shows that the differences between the respondents, such as gender, age, marital status, education status, residence area, having children, lifestyle self-assessment, health self-assessment and having chronic diseases (especially obesity, hypertension, psychiatric diseases), have an impact on the decision to vaccinate. Males, patients with lower education and people living in smaller towns or villages are less likely to get vaccinated. Conversely, people with chronic diseases, those who use health monitoring applications and those who took the flu vaccine have a higher chance of getting vaccinated against COVID-19. This data can be used by doctors, nurses and health care professionals to apply a proper communication strategy to patients in these groups in order to adapt their education and conversation about vaccination against COVID-19. The conclusions can be useful for governments and policy makers to prepare a proper COVID-19 vaccination promotion strategy.

## Figures and Tables

**Table 1:** Participants characteristics.

Variable		Vaccinated against COVID-19 [*n* = 5742, 81.82%]	Unvaccinated against COVID-19 [*n* = 1276, 18.18%]	*p*-Value
**Mean age (SD)**		31 (18–75)	30 (18–73)	**<0.001**
**Gender**	Male [*n* = 1575, 22.44%]	1236 (78.48%)	339 (21.52%)	**<0.001**
	Female [*n* = 5396, 76.89%]	4466 (82.77%)	930 (17.23%)	
	Do not specify [*n* = 47, 0.67%]	40 (85.11%)	7 (14.89%)	
**Marital status**	Married [*n* = 2916, 41.55%	2417 (82.89%)	499 (17.11%)	**0.007**
	Single [*n* = 2054, 29.27%]	1629 (79.31%)	425 (20.69%)	
	Divorced [*n* = 227, 3.23%]	186 (81.94%)	41 (18.06%)	
	Widow [*n* = 13, 0.19%]	9 (69.23%)	4 (30.77%)	
	Cohabitation [*n* = 1808, 25.76%]	1501 (83.02%)	307 (16.98%)	
**Education status**	Higher [*n* = 5087, 72.49%]	4258 (83.70%)	829 (16.30%)	**<0.001**
	Middle [*n* = 1764, 25.14%]	1369 (77.61%)	395 (22.39%)	
	Primary [*n* = 28, 0.40%]	25 (89.29%)	3 (10.71%)	
	Basic vocational [*n* = 60, 0.85%]	35 (58.33%)	25 (41.67%)	
	Lower secondary [*n* = 79, 1.13%]	55 (69.62%)	24 (30.38%)	
**Occupation**	Services [*n* = 5291, 75.39%]	4345 (82.12%)	946 (17.88%)	0.376
	Industry [*n* = 711, 10.13%	574 (80.73%)	137 (19.27%)	
	Agriculture [*n* = 41, 0.58%]	30 (73.17%)	11 (26.83%)	
	Unemployed [*n* = 975, 13.89%]	793 (81.33%)	182 (18.67%)	
**Residence area**	Up to 50,000 [*n* = 925, 13.18%]	722 (78.05%)	203 (21.95%)	**<0.001**
	50,000–150,000 [*n* = 846, 12.05%]	622 (73.52%)	224 (26.48%)	
	150,000–500,000 [*n* = 1158, 16.50%]	941 (81.26%)	217 (18.74%)	
	More than 500,000 [*n* = 3170, 45.17%]	2745 (86.59%)	425 (13.41%)	
	Village [*n* = 919, 13.09%]	712 (77.48%)	207 (22.52%)	
**Children**	Yes [*n* = 2826, 40.27%]	2279 (39,69%)	547 (42,87%)	**0.013**
	No [*n* = 4192, 59.73%]	3463 (60,31%)	729 (57,13%)	
**Overall lifestyle assessment**	Healthy [*n* = 4056, 57.79%]	3221 (79.41%)	835 (20.59%)	**<0.001**
	Unhealthy [*n* = 1136, 16.19%]	981 (86.36%)	155 (13.64%)	
	I do not know [*n* = 1826, 26.02%]	1540 (84.34%)	286 (15.66%)	
**Overall health assessment**	Excellent [*n* = 461, 6.57%]	313 (67.90%)	148 (32.10%)	**<0.001**
	Very good [*n* = 3000, 42.75%]	2393 (79.77%)	607 (20.23%)	
	Good [*n* = 3079, 43.87%]	2642 (85.81%)	437 (14.19%)	
	Not too good [435, 6.20%]	361 (82.99%)	74 (17.01%)	
	Bad [43, 0.61%]	33 (76.74%)	10 (23.26%)	

Data are presented as numbers and percentages. Age is presented in years. Abbreviations: COVID-19, coronavirus disease 19; SD, standard derivation. Significant values (*p* ≤ 0.05) were bolded.

**Table 2:** Participants’ chronic co-morbidities.

Variable	Type of Answer	Vaccinated against COVID-19 [*n* = 5742, 81.82%]	Unvaccinated against COVID-19 [*n* = 1276, 18.18%]	*p*-Value
**Obesity**	Yes [*n* = 785, 11.19%]	689 (87.77%)	96 (12.23%)	**<0.001**
	No [*n* = 6233, 88.81%]	5053 (81.07%)	1180 (18.93%)	
**Diabetes**	Yes [*n* = 125, 1.78%]	95 (76%)	30 (24%)	0.089
	No [*n* = 6893, 98.22%]	5647 (90.60%)	1246 (9.40%)	
**Hypertension**	Yes [*n* = 354, 5.04%]	314 (88.70%)	40 (11.30%)	**<0.001**
	No [*n* = 6664, 94.96%]	5428 (81.45%)	1236 (18.55%)	
**Cancer**	Yes [*n* = 81, 1.15%]	67 (82.72%)	14 (17.28%)	0.833
	No [*n* = 6937, 98.85%]	5675 (81.81%)	1262 (18.19%)	
**Respiratory diseases**	Yes [*n* = 447, 6.37%]	371 (83.00%)	76 (17%)	0.504
	No [*n* = 6571, 93.63%]	5371 (81.74%)	1200 (18.26%)	
**Autoimmune diseases**	Yes [*n* = 1141, 16.26%]	954 (83.61%)	187 (16.39%)	0.086
	No [*n* = 5877, 83.74%]	4788 (81.47%)	1089 (18.53%)	
**Psychiatric diseases**	Yes [*n* = 983, 14.01%]	867 (88.20%)	116 (11.80%)	**<0.001**
	No [*n* = 6035, 85.99%]	4875 (80.78%)	1160 (18.22%)	

Data are presented as numbers and percentages. Abbreviations: COVID-19, coronavirus disease 19. Significant values (*p* ≤ 0.05) were bolded.

**Table 3:** Multivariable logistic regression analysis of factors associated with COVID-19 vaccination.

		Odds Ratio (95% Cl)
**Gender**	Male	**0.83 (0.72–0.97)**
	Do not specify	0.94 (0.41–2.18)
	Female *	1.00
**Age**	>50	0.95 (0.64–1.41)
	40–49	1.12 (0.90–1.41)
	30–39	0.95 (0.81–1.12)
	18–29 *	1.00
**Education status**	Primary	2.23 (0.64–7.73)
	Middle	**0.77 (0.65–0.90)**
	Basic vocational	**0.38 (0.22–0.66)**
	Lower secondary	**0.56 (0.33–0.96)**
	Higher *	1.00
**Marital status**	Cohabitation	1.10 (0.92–1.32)
	Single	0.89 (0.74–1.05)
	Divorced	0.88 (0.60–1.28)
	Widow	0.50 (0.14–1.73)
	Married *	1.00
**Occupation**	Unemployed	**1.30 (1.05–1.59)**
	Industry	1.01 (0.82–1.24)
	Agriculture	0.82 (0.38–1.74)
	Services *	1.00
**Residence area**	150,000–500,000	**0.73 (0.61–0.88)**
	up to 50,000	**0.59 (0.48–0.71)**
	50,000–150,000	**0.47 (0.39–0.57)**
	village	**0.57 (0.47–0.70)**
	more than 500,000 *	1.00
**Chronic comorbidities**	Yes	**1.46 (1.28–1.66)**
	No *	1.00
**Use of health monitoring application**	Yes	**1.56 (1.37–1.77)**
	No *	1.00
**Receive the flu vaccine in 2021 or 2022**	Yes	**24.51 (15.50–38.76)**
	No *	1.00

Data are presented as numbers and percentages. * point of reference based on the largest subgroup Age is presented in years. Abbreviations: COVID-19, coronavirus disease 19; 95% CI, 95% confidence interval. Significant values (*p* ≤ 0.05) were bolded.

## Data Availability

Not applicable.

## Supplementary Materials

The following supporting information can be downloaded at: https://www.mdpi.com/article/10.3390/vaccines10101715/s1, S1: Questionnaire form; S2: Multivariable logistic regression analysis of factors associated with COVIDd-19 vaccination, Table S1: Multivariable logistic regression analysis of factors associated with COVID-19 vaccination.
