# Supplementary material for: Factors Associated with Willingness to Receive a COVID-19 Vaccine in Adult Polish Population—A Cross-Sectional Survey
Source: Vaccines (Basel). 2022 Oct 14;10(10):1715. doi: 10.3390/vaccines10101715 (PMC9609836; doi:10.3390/vaccines10101715)
Supplement: Supplementary file 1 [file vaccines-10-01715-s001.zip › vaccines-1918872-supplementary.pdf]

Article

Factors Associated With Willingness To Receive a COVID-19 Vaccine in Adult Polish Population – Cross-Sectional Survey

Katarzyna Ulaszewska [1], Alicja Monika Jodczyk [1], Piotr Długołęcki [2], Sara Emerla [1,†], Wiktoria Stańska [1,†], Przemysław Seweryn Kasiak [1*], Jakub S. Gąsior [4], Damian Parol [5], Artur Mamcarz [2,5], and Daniel Śliż [1,2,3,5]

1 Students' Scientific Group of Lifestyle Medicine, 3rd Department of Internal Medicine and Cardiology, Medical University of Warsaw, 04-749 Warsaw, Poland;
2 Polish Society of Lifestyle Medicine, 00-388 Warsaw, Poland,
3 School of Public Health, Postgraduate Medical Education Center, 01-813 Warsaw, Poland,
4 Department of Pediatric Cardiology and General Pediatrics, Medical University of Warsaw, 02-091 Warsaw, Poland
5 3rd Department of Internal Diseases and Cardiology, Medical University of Warsaw, 04-749 Warsaw, Poland
* Correspondence: to whom correspondence should be addressed.
Correspondence: przemyslaw.kasiak1@gmail.com tel,: +48-501168103, .
† Equally contributed as fourth authors.

Supplementary Material 1: Questionnaire form

ENGLISH VERSION

Part 1. General and demographic questions

- Age
  _____ (in years from 18 to 100)

- Gender
  _____ (male/female/do not specify)

- Marital status
  _____ (married/single/divorced/widow/cohabitation)

- Education status
  _____ (higher/middle/primary/basic vocational/lower secondary)

- Occupation field
  _____ (services/industry/agriculture/unemployed)

- Residence area
  _____ (urban – up to 50 000/urban – 50 000–150 000 inhabitants/urban – 150 000–500 000 inhabitants/urban – more than 500 000 inhabitants/village)

- How many children do you have?
  _____ (0/1/2/3/4/5/6/7/8/9/10 or more)

Part 2. Health and lifestyle

- How would you rate your lifestyle?
  _____ (healthy/unhealthy/I do not know)

- How would you rate your health?
  _____ (excellent/very good/good/not too good/bad)

- Do you have any of the chronic diseases listed below, or any others?
  - Obesity _____ (yes/no)
  - Diabetes _____ (yes/no)
  - Hypertension _____ (yes/no)
  - Cancer _____ (yes/no)
  - Respiratory diseases _____ (yes/no)
  - Autoimmune diseases _____ (yes/no)

- Psychiatric diseases _____ (yes/no)
- Arteriosclerosis _____ (yes/no)
- Other CVDs _____ (yes/no)
- Digestive system diseases _____ (yes/no)
- Musculoskeletal system diseases _____ (yes/no)
- Neurological diseases _____ (yes/no)
- Other _____ (yes/no)

- Do you use apps to monitor and improve your health?
  (e.g. counting steps, calories, workout time)
  _____ (yes/no)

- Did you get a flu vaccination in 2021 or 2022?
  _____ (yes/no)

<u>Part 3. Vaccination against COVID-19</u>

- Have you been vaccinated against COVID-19?
  _____ (yes/no)

POLISH Version

Część 1. Podstawowe dane osobowe

- Wiek
  _____ (w latach od 18 to 100)

- Płeć
  _____ (mężczyzna/kobieta/wolę nie podawać)

- Stan cywilny
  _____ (w związku małżeńskim/panna, kawaler/rozwódka, rozwodnik/wdowa, wdowiec/w nieformalnym związku)

- Wykształcenie
  _____ (wyższe/średnie/podstawowe/zasadnicze zawodowe/ gimnazjalne)

- Rodzaj wykonywanej pracy
  _____ (usługi/przemysł/rolnictwo/bezrobotny)

- Miejsce zamieszkania
  _____ (miasto do 50 tys. mieszkańców/miasto 50-100 tys. mieszkańców/miasto 100-500 tys. mieszkańców/miasto powyżej 500 tys. mieszkańców/wieś)

- Proszę zaznaczyć ile posiada Pan/Pani dzieci?
  _____ (0/1/2/3/4/5/6/7/8/9/10 lub więcej)

Część 2. Zdrowie i styl życia

- Czy uważa się Pan/Pani za osobę, która prowadzi zdrowy styl życia?
  _____ (tak/nie/trudno powiedzieć)

- Jakby Pan/Pani ocenił/a swoje zdrowie?
  _____ (doskonałe/bardzo dobre/dobre/niezbyt dobre/złe)

- Czy choruje Pan/Pani na choroby przewlekłe wymienione poniżej, lub inne?
  - Otyłość _____ (tak/nie)
  - Cukrzyca _____ (tak/nie)
  - Nadciśnienie _____ (tak/nie)
  - Nowotwory _____ (tak/nie)
  - Choroby układu oddechowego _____ (tak/nie)
  - Choroby autoimmunologiczne _____ (tak/nie)
  - Choroby psychiatryczne _____ (tak/nie)

    - Miażdżyca _____ (tak/nie)
    - Inne choroby układu krążenia _____ (tak/nie)
    - Choroby układu pokarmowego _____ (tak/nie)
    - Choroby układu ruchu _____ (tak/nie)
    - Choroby neurologiczne _____ (tak/nie)
    - Inne _____ (tak/nie)

- Czy korzysta Pan/Pani z aplikacji mających na celu kontrolę i poprawę stanu zdrowia?
  (n.p. liczące kroki, kalorie, czas treningu)
  _____ (tak/nie)

- Czy w roku 2021 lub 2022 zaszczepił/a się Pan/Pani przeciw grypie?
  _____ (tak/nie)

<u>Part 3. Decyzja o zaszczepieniu przeciwko COVID-19</u>

- Czy zaszczepił/a się Pan/Pani przeciw COVID-19?
  _____ (tak/nie)

Supplementary Material 2: Multivariable logistic regression analysis of factors associated with COVID-19 vaccination

Table S1. Multivariable logistic regression analysis of factors associated with COVID-19 vaccination.

**Variables**	Categories	β	Wald stat. 95% CI	Odds ratio (95% CI)	p-Value
**Gender**	Male			0.84	0.025
	Do not specify			1.06	0.889
	Female				
**Age**	>50			1.35	0.116
	40-49			1.38	0.004
	30-39			1.03	0.687
	18-29				
**Education status**	Primary			1.94	0.289
	Middle			0.71	<0.000
	Basic vocational			0.33	<0.000
	Lower secondary			0.50	0.009
	Higher				
**Marital status**	Cohabitation			1.04	0.675
	Single			0.85	0.053
	Divorced			0.84	0.333
	Widow			0.32	0.068
	Married				
**Occupation**	Unemployed			1.36	0.003
	Industry			0.99	0.887
	Agriculture			0.82	0.584
	Services				
**Residence area**	150 000–500 000			0.70	<0.000
	50 000–150 000			0.44	<0.000
	up to 50 000			0.56	<0.000
	village			0.55	<0.000
	more than 500 000				
**Chronic comorbidities**	Yes			1.53	<0.000
**Use of health monitoring application**	Yes			1.56	<0.000

The Hosmer-Lemeshow goodness-of-fit test produced a test statistic of 7.569 (with a p-value of 0.477). AUC of the regression model was 0.644.
